# Malaria Control Interventions Contributed to Declines in Malaria Parasitemia, Severe Anemia, and All-Cause Mortality in Children Less Than 5 Years of Age in Malawi, 2000–2010

**DOI:** 10.4269/ajtmh.17-0203

**Published:** 2017-09-27

**Authors:** Christine L. Hershey, Lia S. Florey, Doreen Ali, Adam Bennett, Misheck Luhanga, Don P. Mathanga, S. René Salgado, Carrie F. Nielsen, Peter Troell, Gomezgani Jenda, Yazoume Yé, Achuyt Bhattarai

**Affiliations:** 1 President’s Malaria Initiative, Agency for International Development, Washington, District of Columbia;; 2 The DHS Program, ICF International, Rockville, Maryland;; 3 National Malaria Control Program, Lilongwe, Malawi;; 4 Global Health Group, University of California San Francisco School of Medicine, San Francisco, California;; 5 Malaria Alert Centre, College of Medicine, Blantyre, Malawi;; 6 President’s Malaria Initiative, Malaria Branch, Centers for Disease Control and Prevention, Atlanta, Georgia;; 7 President’s Malaria Initiative, Centers for Disease Control and Prevention, Lilongwe, Malawi;; 8 President’s Malaria Initiative, Agency for International Development, Lilongwe, Malawi;; 9 MEASURE Evaluation, ICF International, Rockville, Maryland

## Abstract

Malaria control intervention coverage increased nationwide in Malawi during 2000–2010. Trends in intervention coverage were assessed against trends in malaria parasite prevalence, severe anemia (hemoglobin < 8 g/dL), and all-cause mortality in children under 5 years of age (ACCM) using nationally representative household surveys. Associations between insecticide-treated net (ITN) ownership, malaria morbidity, and ACCM were also assessed. Household ITN ownership increased from 27.4% (95% confidence interval [CI] = 25.9–29.0) in 2004 to 56.8% (95% CI = 55.6–58.1) in 2010. Similarly intermittent preventive treatment during pregnancy coverage increased from 28.2% (95% CI = 26.7–29.8) in 2000 to 55.0% (95% CI = 53.4–56.6) in 2010. Malaria parasite prevalence decreased significantly from 60.5% (95% CI = 53.0–68.0) in 2001 to 20.4% (95% CI = 15.7–25.1) in 2009 in children aged 6–35 months. Severe anemia prevalence decreased from 20.4% (95% CI: 17.3–24.0) in 2004 to 13.1% (95% CI = 11.0–15.4) in 2010 in children aged 6–23 months. ACCM decreased 41%, from 188.6 deaths per 1,000 live births (95% CI = 179.1–198.0) during 1996–2000, to 112.1 deaths per 1,000 live births (95% CI = 105.8–118.5) during 2006–2010. When controlling for other covariates in random effects logistic regression models, household ITN ownership was protective against malaria parasitemia in children (odds ratio [OR] = 0.81, 95% CI = 0.72–0.92) and severe anemia (OR = 0.82, 95% CI = 0.72–0.94). After considering the magnitude of changes in malaria intervention coverage and nonmalaria factors, and given the contribution of malaria to all-cause mortality in malaria-endemic countries, the substantial increase in malaria control interventions likely improved child survival in Malawi during 2000–2010.

## INTRODUCTION

Malaria caused by *Plasmodium falciparum* is endemic throughout Malawi.^1^ Malaria transmission is stable with seasonal peaks related to the rainfall during November to April.^2^ The Ministry of Health (MoH) in Malawi reported that malaria accounted for 40% of all outpatient visits and 40% of hospital deaths in 2001, and was the cause of workforce losses (up to 25 days of work lost per year on average).^3^ Malaria was also the leading cause (39%) of hospital admissions in children less than 5 years of age.^3^

There have been substantial increases in funding for malaria control in Malawi that started around 2006 and which came mainly from the Global Fund to Fight AIDS, Tuberculosis and Malaria (Global Fund) and the President’s Malaria Initiative, in total amounts of $42.2 and $83.1 million, respectively, from 2006 to 2010.^4^ The Malawi Government and other funding partners contributed funding for malaria control as well during this time.^5^ The Malawi Government and its national and international partners have shown a strong continued commitment to malaria control and prevention, which is reflected in three successive 5-year Malaria Strategic Plans since 2000.^3,6,7^

**Figure 1.**
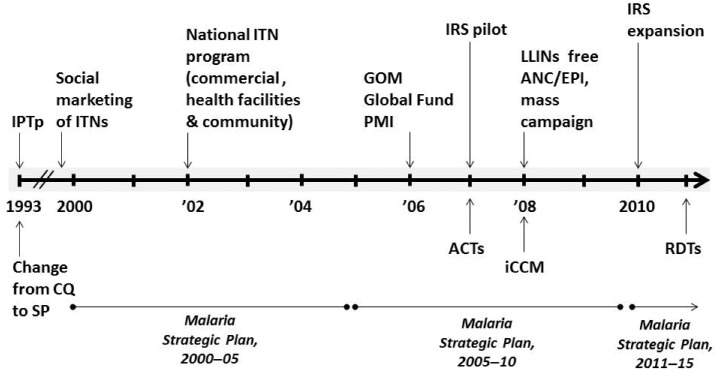
Timeline of milestones in Malawi’s malaria control program, 1993–2011. The periods covered by the three Malaria Strategic Plans are shown, along with changes in the first-line antimalarial drugs (CQ, SP and, ACTs) and implementation of malaria control interventions. iCCM was introduced in 2008 and expanded thereafter.^12^ Rapid diagnostic tests (RDTs) were introduced after the 2000–2010 evaluation period. CQ = chloroquine; SP = sulfadoxine-pyrimethamine; ACTs = artemisinin-based combination therapies; IPTp = intermittent preventive treatment in pregnancy; IRS = indoor residual spraying; ITNs = insecticide treated nets; ANC = antenatal care; EPI = expanded program on immunizations; iCCM = integrated community case management; GOM = Government of Malawi; PMI = President’s Malaria Initiative.

Malawi has been at the forefront of the introduction of malaria control interventions in sub-Saharan Africa. The country initially used social marketing (where individuals contribute to the cost by paying for subsidized nets or insecticides)^8^ and the commercial sector to deliver insecticide-treated nets (ITNs) and had the first national ITN program in sub-Saharan Africa by 2003.^7^ The Malawi MoH has recommended using sulfadoxine-pyrimethamine (SP) for malaria intermittent preventive treatment during pregnancy (IPTp) since 1993,^9,10^ long before the World Health Organization (WHO) recommended IPTp in 2002.^5^ Over the years, the Malawi Government changed malaria treatment policies in response to the emergence of antimalarial drug resistance. In 1993, Malawi was the first country in sub-Saharan Africa to replace chloroquine with SP as the first-line treatment of uncomplicated malaria. In 2007, Malawi changed from SP as the recommended first-line antimalarial to artemether-lumefantrine (AL), an artemisinin-based combination therapy, following significant evidence of malaria parasite resistance to SP.^11^ These key milestones are summarized in Figure 1.

Given the increased investments, changes in malaria control polices, and early adoption of interventions in Malawi, this study evaluated the impact of malaria control efforts on malaria morbidity and all-cause childhood mortality (ACCM) during 2000–2010.

## MATERIALS AND METHODS

### Evaluation design.

**Figure 2.**
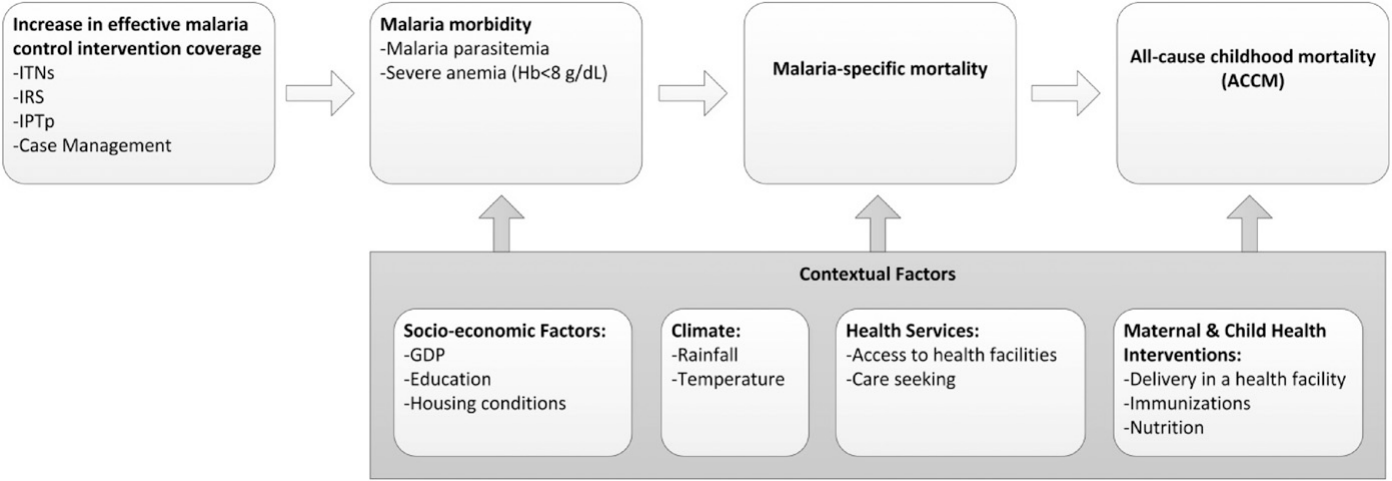
Conceptual framework. The plausibility analysis framework is based on an increase in coverage of effective malaria control interventions leading to lower malaria morbidity and lower malaria mortality, which in highly endemic settings would result in lowering ACCM.^13–15^ The analysis accounts for other contextual factors that may affect malaria morbidity, malaria mortality, or ACCM. See Yé and others^15^ for a more detailed explanation of the conceptual framework. Examples of contextual factors are shown, but this is not an exhaustive list. Hb = hemoglobin; ACCM = all-cause childhood mortality.

The evaluation of the impact of malaria control efforts in Malawi was based on a before-and-after plausibility assessment, as recommended by the Roll Back Malaria (RBM) Partnership’s Monitoring and Evaluation Reference Group (MERG) (Figure 2).^13^ The rationale for using this evaluation design with ACCM as the primary impact measure is discussed in depth elsewhere.^13–15^ The RBM MERG recommended using ACCM as a primary impact measure where baseline transmission intensity is moderate or high (i.e., entomological inoculation rate [EIR] > 10 infective bites/person/year or childhood parasite prevalence is > 25%).^13^ This evaluation design was strengthened by using logistic regression models to assess the strength of association between household ITN ownership and malaria parasitemia and severe anemia among child household members during the evaluation period, two malaria morbidity measures which are in the causal pathway between malaria control interventions and changes in ACCM (Figure 2). Multivariable regression models were also used to evaluate the association between household ITN ownership and ACCM under programmatic conditions which are separately reported by Florey and others^16^ in this journal supplement.

### Data sources and evaluation indicators.

#### Nationally representative household surveys.

A list of population-based evaluation indicators and data sources are shown in Supplemental Table 1. The Demographic and Health Survey (DHS), Multiple Indicator Cluster Survey (MICS), and Malaria Indicator Survey (MIS) use a two-stage survey design, typically with the most recent census as the sampling frame for enumeration units. The DHS Program^17^ and United Nations Children’s Fund MICS^18^ websites and survey reports provide more detailed information on sampling of these surveys. The 2010 DHS results for intervention coverage and severe anemia were used for trend analysis instead of the 2010 MIS results due to seasonal comparability with the other surveys used in this evaluation. DHS field work was conducted in the low malaria transmission season and the MIS in the high malaria transmission season.

**Table 1:** Household attributes, asset ownership, and women’s* education, Malawi, 2000 and 2010

Indicators	2000			2010			
	%	95% CI	WN	%	95% CI	WN	Relative change (%)
Improved water source (protected, borehole, piped) (% households)	65.2	62.1–68.2	14,213	79.7	77.7–81.5	24,825	22.2
Improved roof (not thatch/grass/mud) (% households)†	25.8	24.5–27.3	30,553	35.0	32.9–37.0	24,825	35.7
Electricity (% households)	4.8	3.6–6.4	14,213	8.7	7.6–9.9	24,825	81.3
Telephone (landline or mobile) (% households)‡	5.1	3.8–6.9	13,664	39.3	37.6–41.1	24,825	670.6
Completed primary education (% women 15–49)	19.1	17.1–21.2	13,220	29.3	27.8–30.8	23,020	53.4
Literacy (% women 15–49)	56.5	54.5–58.4	13,220	67.6	66.3–69.0	23,020	19.6

CI = confidence interval; DHS = Demographic and Health Survey; MICS = Multiple Indicator Cluster Survey; WN = weighted number of units (denominator).

* Women aged 15–49 years. Data are from the 2000 and 2010 DHS surveys unless otherwise noted.

† 2006 MICS source.

‡ 2004 DHS source for the baseline estimate. Sample sizes vary based on the population of interest corresponding to each indicator.

We calculated the proportion of women with a live birth 0–2 years before the survey who received at least two doses of SP for IPTp. Data on the source of SP medications were only collected in the 2004 and 2010 DHS, so source was not included in the calculation of this indicator.

Severe anemia prevalence (hemoglobin concentration < 8 g/dL)^19^ in children aged 6–59 months who slept in a sampled household the night before the survey was calculated using DHS data. Hemoglobin values were adjusted for altitude according to the standard methodology used by the DHS.^20,21^

The National Micronutrient Surveys (NMSs) are nationally representative cross-sectional household surveys, in which data were collected on severe anemia (Hb < 8 g/dL) and parasitemia (microscopy-based) in children 6–35 months of age in 2001 and 2009.^22,23^ The 2001 NMS was sampled for children 6–35 months old, whereas the 2009 survey was sampled for children 6–59 months old. The 2009 survey was reanalyzed to generate estimates for children 6–35 months of age for comparison purposes. The NMS were conducted in the low transmission season. Malaria parasite prevalence (microscopy-based) data were also obtained for children 6–59 months of age from the 2010 MIS (field work March to April 2010) during the peak malaria transmission season.

### Sub-national survey data.

Sub-national data on severe anemia (Hb < 8 g/dL) and malaria parasite prevalence estimates were obtained from anemia and parasitemia surveys,^24^ and used in logistic regression models to assess the association between ITN ownership and parasitemia or severe anemia. These surveys measured malaria parasitemia (by microscopy) and severe anemia in children 6–30 months of age in six districts in 2005 and 2006 and in eight districts in 2007, 2008, and 2009. These surveys were conducted in April of each year, which represents the latter portion of the rainy season and therefore the peak of the malaria transmission season. The six districts surveyed in 2005 and 2006 included four from Southern Region (Chiradzulu, Mwanza, Phalombe, and Blantyre), and one each from Central Region (Lilongwe) and Northern Region (Rumphi). The 2006 parasitemia data were not available for this study and therefore 2006 data were not included in the logistic regression models discussed below. In 2007, 2008, and 2009, an additional district was sampled in both the Central Region (Nkhotakota) and Northern Region (Karonga).

### Contextual factor data.

Contextual factor data including information on household attributes, asset ownership, women’s education, human immunodeficiency virus (HIV) prevalence, and maternal and child health interventions were obtained from the DHS, MICS, and MIS surveys unless otherwise indicated. Gross domestic product (GDP) per capita purchasing power parity (PPP) data were obtained from the World Bank (http://data.worldbank.org/country/malawi).^25^

Temperature data were obtained from the NASA Land Processes Distributed Active Archive Center Data Pool from the United States Geological Survey (USGS)/Earth Resources Observation and Science Center, Moderate Resolution Imaging Spectroradiometer satellite.^26^ Rainfall data were available from the USGS Famine Early Warning System Network data portal.^27^ These data were used in the logistic regression models described below. Additional analysis of meteorological data for the Malawi evaluation is included in Thomson and others in this journal supplement.^28^

### Data analysis and statistical methods.

Five-year all-cause mortality rates were calculated from the 2000, 2004, and 2010 DHS using a synthetic cohort life table approach described in detail in the Guide to DHS Statistics.^21^ Mortality rates were not calculated from the 2006 MICS because these 5-year estimates overlap with the estimates from the 2004 and 2010 DHS surveys making it difficult to detect changes between the surveys. ACCM estimates were stratified by urban and rural residence (as defined by DHS) and malaria risk zones. Malaria risk zones were determined by overlaying the cluster locations from the 2010 DHS on a 2007 map of estimated prevalence rates of *P. falciparum* infection in children 2–10 years of age (*Pf*PR_2–10_) from the Malaria Atlas Project.^29^ Terciles of *Pf*PR_2–10_ values were then created at the cluster level. Malaria parasite prevalence ranges for the three risk categories included 0–33.7% (lower); 33.8–45.2% (medium); and 45.3–100% (higher). Disaggregated annual estimates of ACCM with 95% confidence intervals (CIs), for the 1990–2010 period were calculated from the 2000 (1991–1999) and 2010 (2000–2010) DHS data following a similar method as for the 5-year mortality rates, but limited to 1-year intervals.

Malaria control intervention coverage and severe anemia prevalence estimates were stratified by sex, residence (urban/rural), wealth quintiles, and mother’s education (none, primary, secondary, or higher). Severe anemia prevalence was disaggregated by malaria risk terciles and by age groups (6–23 months and 24–59 months), as studies have shown the impact of malaria control interventions on malaria morbidity in these age groups to be different in malaria-endemic countries.^30–32^

Statistical significance for differences was determined by assessing the 95% CIs around each of the survey estimates. Nonoverlapping CIs for estimates from two survey years were deemed statistically significant. All analyses were performed using Stata 12 (StataCorp LP, College Station, TX).

Separate multivariable, random effects logistic regression models from the sub-national survey data predicting the outcomes of malaria parasitemia and severe anemia were conducted using household ITN ownership, defined as at least one ITN in the household, as the primary ITN exposure variable. Although the proportion of children sleeping under an ITN might have been used as an indicator of ITN exposure, it has been shown that the pooled effect of children sleeping under an ITN the previous night or household ITN ownership on the prevalence of parasitemia are of similar magnitude and not significantly different.^33^ The models included a random intercept for each enumeration area. The models assessed the odds of malaria parasitemia or severe anemia for children aged 6–30 months after controlling for survey year and child, household, region, and meteorological factors. Differences in meteorological factors (at the district level) preceding each survey (sub-national surveys were conducted in April) were controlled for by including anomalies from the 5-year means for February and March rainfall and minimum temperature. Districts were collapsed into regional categories (Northern, Central, and Southern). An interaction term for survey year and region was included in the models. Malaria infection (malaria parasite positive by microscopy) was included as a covariate in the severe anemia model.

## RESULTS

### ITNs and indoor residual spraying.

**Figure 3.**
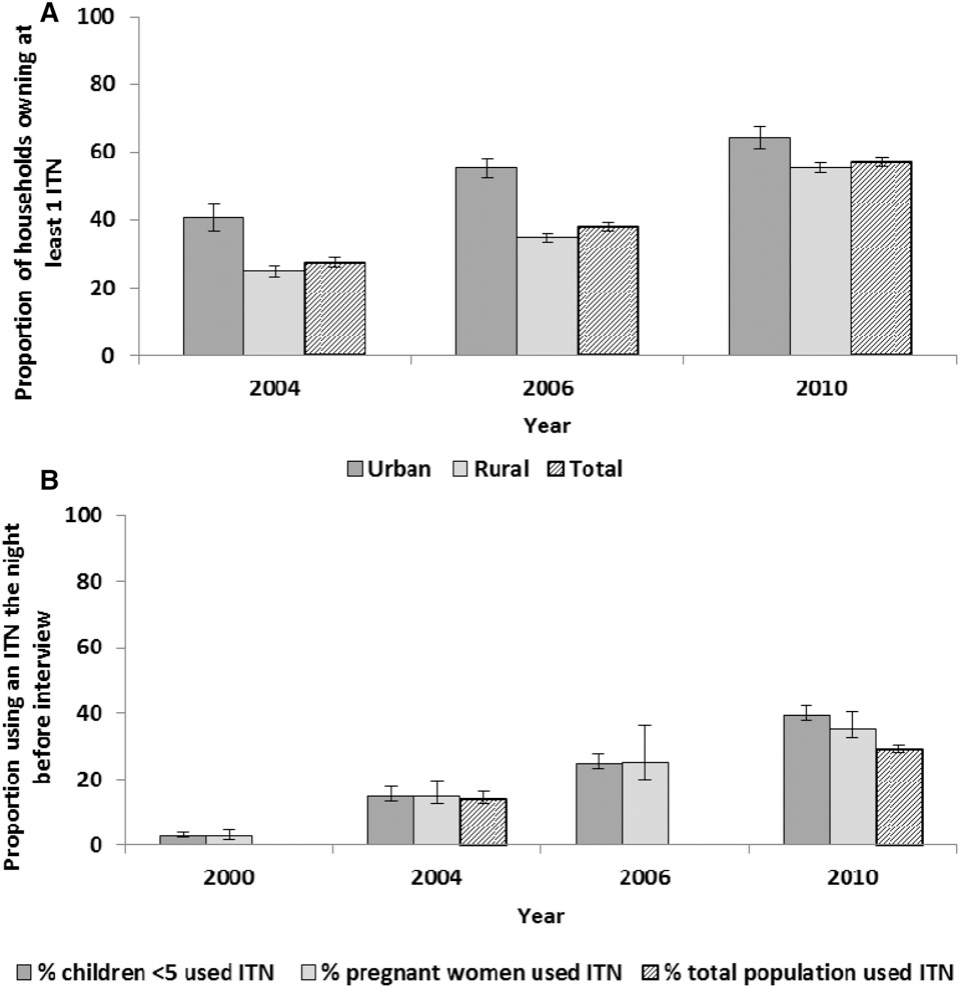
Household ownership of insecticide treated nets (ITNs) and use of ITNs, Malawi, 2000–2010. (**A**) Household ownership of at least one ITN is shown for all households and disaggregated by urban/rural residence. Questions on brand of net and on treatment of nets with insecticide were not included in the 2000 Demographic and Health Survey (DHS); thus, ITN ownership cannot be estimated for this survey. (**B**) Use of an ITN is estimated for children less than 5 years of age, pregnant women, and the entire population in the household the night before the survey. Questions on net use by the total population were not included in the 2000 DHS or the 2006 Multiple Indicator Cluster Survey (MICS). The 2006 MICS is only representative for pregnant women who have had a birth in the past 2 years.

Household ownership of at least one ITN rose significantly from 27.4% (95% CI = 25.7–29.0) in 2004 to 56.8% (95% CI = 55.6–58.1) in 2010 (Figure 3A). Household ownership of ITNs varied greatly by location of household residence with urban households being significantly more likely to own at least one ITN compared with rural households in all survey years, although this disparity appears to have narrowed by 2010 (Figure 3A). Although some geographic variation in household ownership of ITNs existed at the district level, ITN ownership improved in all districts in 2010 as compared with 2004 (see Supplemental Figure 1 for maps of district-level household ITN ownership).

Similar to household ITN ownership, ITN use (sleeping under an ITN the night before the survey) increased substantially between 2000 and 2010 (Figure 3B). ITN use the night before the survey among all children less than 5 years of age increased from 2.8% (95% CI = 2.3–3.1) in 2000 to 39.4% (95% CI = 38.0–40.8) in 2010 (Figure 3B). Similarly, ITN use by pregnant women increased from 2.6% (95% CI = 1.8–3.8) in 2000 to 35.2% (95% CI = 32.6–37.9) in 2010 (Figure 3B). ITN use increased for both children under five and pregnant women across residence (urban/rural), wealth, and education strata (Supplemental Tables 2 and 3). ITN use also improved among the total population, increasing from 13.8% (95% CI = 12.6–15.1) in 2004 to 29.0% (95% CI = 28.0–30.1) in 2010 (Figure 3B).

Indoor residual spraying (IRS) was implemented sub-nationally in Malawi. Starting as a pilot in 2007, it was then expanded to seven districts along Lake Malawi and in the Shire Valley. At the time of the 2010 DHS, only households in Nkhotakota District had been sprayed during the year, resulting in 59.4% of the households in Nkhotakota District having been sprayed with IRS in the past 12 months (2.2% nationally).

### Intermittent preventive treatment in pregnancy.

**Figure 4.**
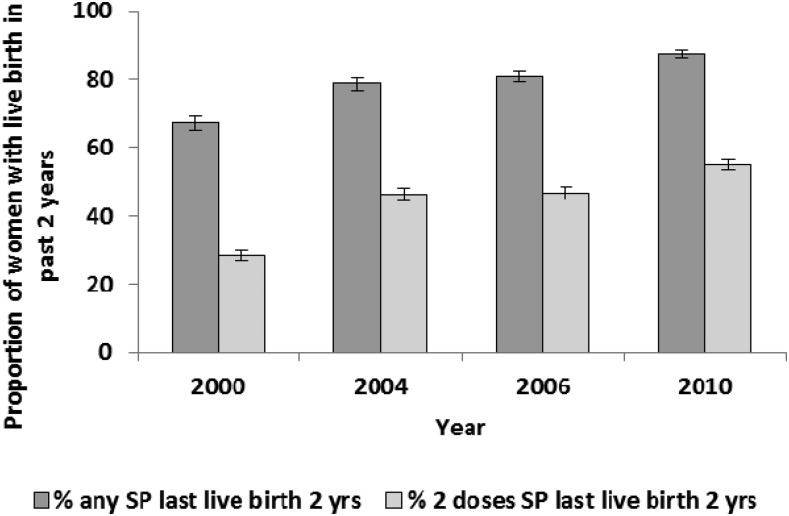
Proportion of women receiving sulfadoxine-pyrimethamine (SP) for prevention of malaria in pregnancy, Malawi, 2000–2010. Estimates are shown for women (15–49 years) with a live birth in the last 2 years who took one or two doses of SP for malaria prevention during their last pregnancy, regardless of the source of the medication.

The proportion of women with a live birth in the 2 years preceding the survey who received at least two doses of SP during pregnancy increased from 28.2% (95% CI = 26.7–29.8) to 55.0% (95% CI = 53.4–56.6) between 2000 and 2010 (Figure 4). IPTp coverage increased in both urban and rural residences, in all strata of women’s educational attainment, and in all wealth strata (Supplemental Table 4).

### Case management of malaria.

**Figure 5.**
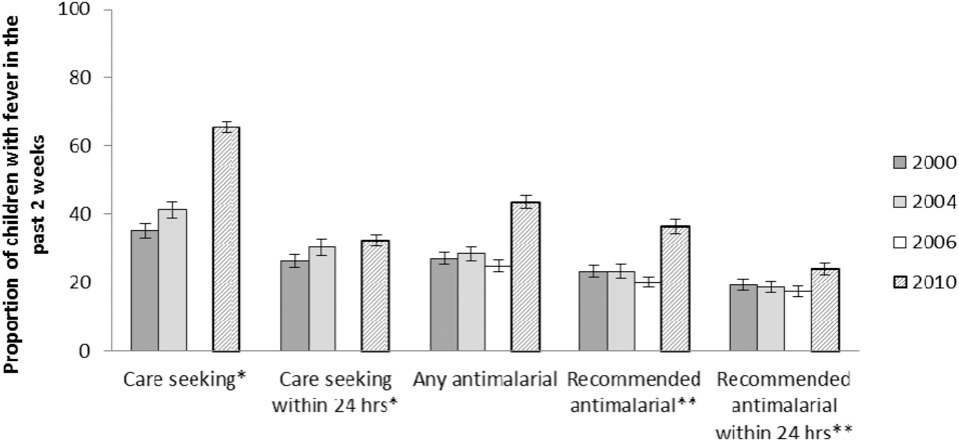
Percentage of children less than 5 years of age with fever who sought care and were treated with antimalarial drugs, Malawi, 2000–2010. Caregivers of children under five with a fever in the 2 weeks before the Demographic and Health Survey and Multiple Indicator Cluster Survey (MICS) interview were asked about the care seeking and treatment their child received for the fever. *Care seeking refers to seeking advice or treatment of fever from a public or private health professional or from a pharmacy, including health surveillance assistants/community health workers, but excluding shops and traditional healers. Care seeking was not included in the 2006 MICS. **The recommended first-line antimalarial was sulfadoxine-pyrimethamine in 2000, 2004, and 2006 and artemisinin-based combination therapy (artemether-lumefantrine) in 2010.

Although overall care seeking for fever in children under five has increased over the decade from 35.3% (95% CI = 33.1–37.5) to 65.4% (95% CI = 63.6–67.1), timely care seeking (within 24 hours of fever onset) has increased only slightly, albeit a statistically significant change (26.4% [95% CI = 24.6–28.3] in 2000 and 32.3% [95% CI = 30.7–34.0] in 2010) (Figure 5). Care seeking for fever improved across sex, residence, wealth, and education strata (Supplemental Table 5). Of all children less than 5 years of age who experienced fever in the prior 2 weeks, use of any antimalarial increased over the study period from 27.1% (95% CI = 25.3–28.9) in 2000 to 43.4% (95% CI = 41.4–45.4) in 2010. The proportion of febrile children treated with a recommended (first-line) antimalarial drug did not change between 2000 (23.3% [95% CI = 21.6–25.0]) and 2006 (20.2% [95% CI = 18.7–21.8]); however, an increase occurred between 2006 and 2010 (36.4% [95% CI = 34.4–38.4]) (Figure 5). There was a slight, but significant, change in prompt (within 24 hours) use of a first-line antimalarial, increasing from 19.4% (95% CI = 17.7–21.2) in 2000 to 24.1% (95% CI = 22.5–25.7) in 2010. Of children with fever who received antimalarial treatment, over 80% were given the currently recommended first-line medication in all survey years between 2000 and 2010 (Supplemental Table 6). SP was the recommended first-line antimalarial in 2000, 2004, and 2006 and ACTs (AL) in 2010.

### Malaria morbidity.

**Figure 6.**
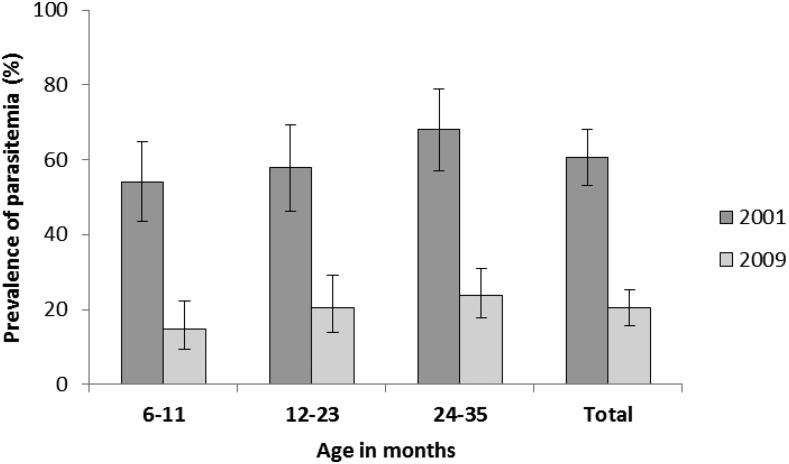
Malaria parasite prevalence in children 6–35 months by age, Malawi, 2001 and 2009. Parasitemia was measured via microscopy in the National Micronutrient Surveys in 2001 and 2009. The 2001 survey was conducted September to October and the 2009 survey was conducted July to August, both in the low malaria transmission season. Parasitemia estimates are shown for all children aged 6–35 months (total) and also disaggregated into smaller age categories: 6–11, 12–23, and 24–35 months.

In the Malawi NMSs, conducted during the low transmission season, malaria parasite prevalence in children 6–35 months of age decreased from 60.5% (95% CI = 53.0–68.0) in 2001 to 20.4% (95% CI = 15.7–25.1) in 2009 (Figure 6). Malaria parasite prevalence in children 6–59 months of age was 43.3% (95% CI = 38.6–48.0) by microscopy in the 2010 MIS. No other MIS was conducted during the evaluation period.

**Figure 7.**
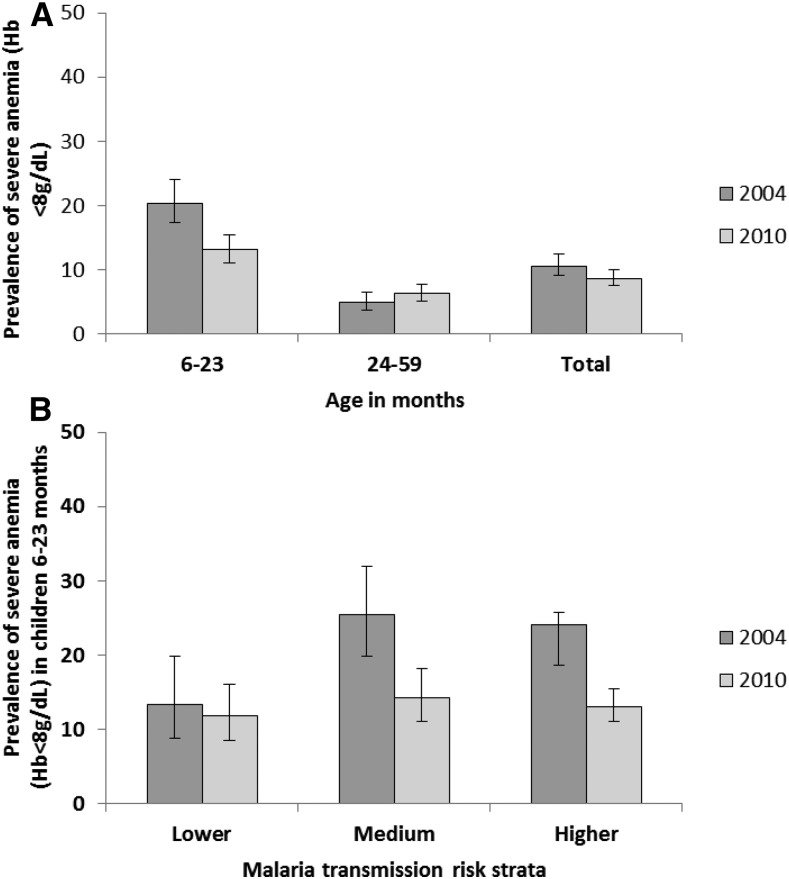
Trends in severe anemia (hemoglobin [Hb] < 8 g/dL) prevalence by age and malaria risk areas, Malawi, 2004 and 2010. (**A**) Prevalence of severe anemia (Hb < 8 g/dL) was measured in children 6–59 months of age (total). This was also disaggregated into children 6–23 months of age and 24–59 months of age. (**B**) Severe anemia prevalence in children 6–23 months of age was further disaggregated by malaria transmission risk zones. (See Materials and Methods for a description of the risk stratification). Data are from the 2004 and 2010 Demographic and Health Surveys.

The prevalence of severe anemia did not decrease significantly between 2004 (10.6% [95% CI = 9.1–12.4]) and 2010 (8.7% [95% CI = 7.6–10.0]) in children aged 6–59 months using the DHS surveys. However, significant decreases were evident in subgroups of younger children, who were at higher risk for severe anemia than older children. Severe anemia prevalence in children 6–23 months of age declined from 20.4% (95% CI = 17.3–24.0) in 2004 to 13.1% (95% CI = 11.0–15.4) in 2010, a relative change of 36% (Figure 7A), whereas in older children 24–59 months of age no decline was observed (5.0% [95% CI = 3.8–6.5] in 2004 and 6.3% [95% CI = 5.1–7.8] in 2010). The prevalence of severe anemia in children 6–23 months declined markedly in areas of medium (43.9% relative decline) and higher (45.6% relative decline) malaria transmission, but less so in areas of lower malaria transmission (11.9% relative decline) using DHS data (Figure 7B). Severe anemia declined in rural, but not urban areas between 2004 and 2010, but the changes were not significant (Supplemental Table 7). Declines in severe anemia were also seen in the NMS, where severe anemia in children 6–35 months declined significantly from 17.7% (95% CI = 13.5–21.8) in 2001 to 7.3% (95% CI = 4.8–9.8) in 2009 (Supplemental Figure 2).

### Mortality.

**Figure 8.**
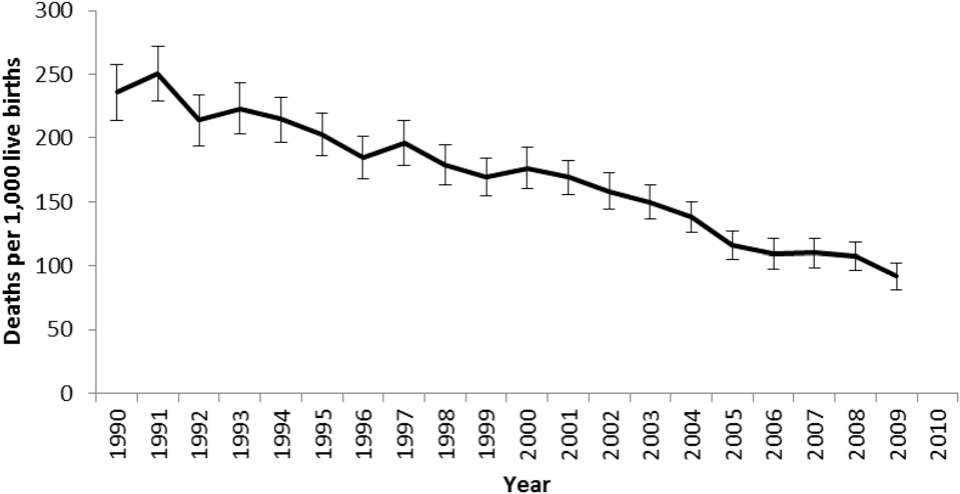
Disaggregated annual all-cause under-five mortality, Malawi, 1990–2010. Annual estimates were calculated for all-cause under-five mortality from 1990 to 2010 using the 2000 and 2010 Demographic and Health Surveys. For comparisons with the United Nations Interagency Group for Child Mortality Estimation^34^ and Institute for Health Metrics and Evaluation^35^ annual estimates of under-five mortality see Supplemental Figure 3.

**Figure 9.**
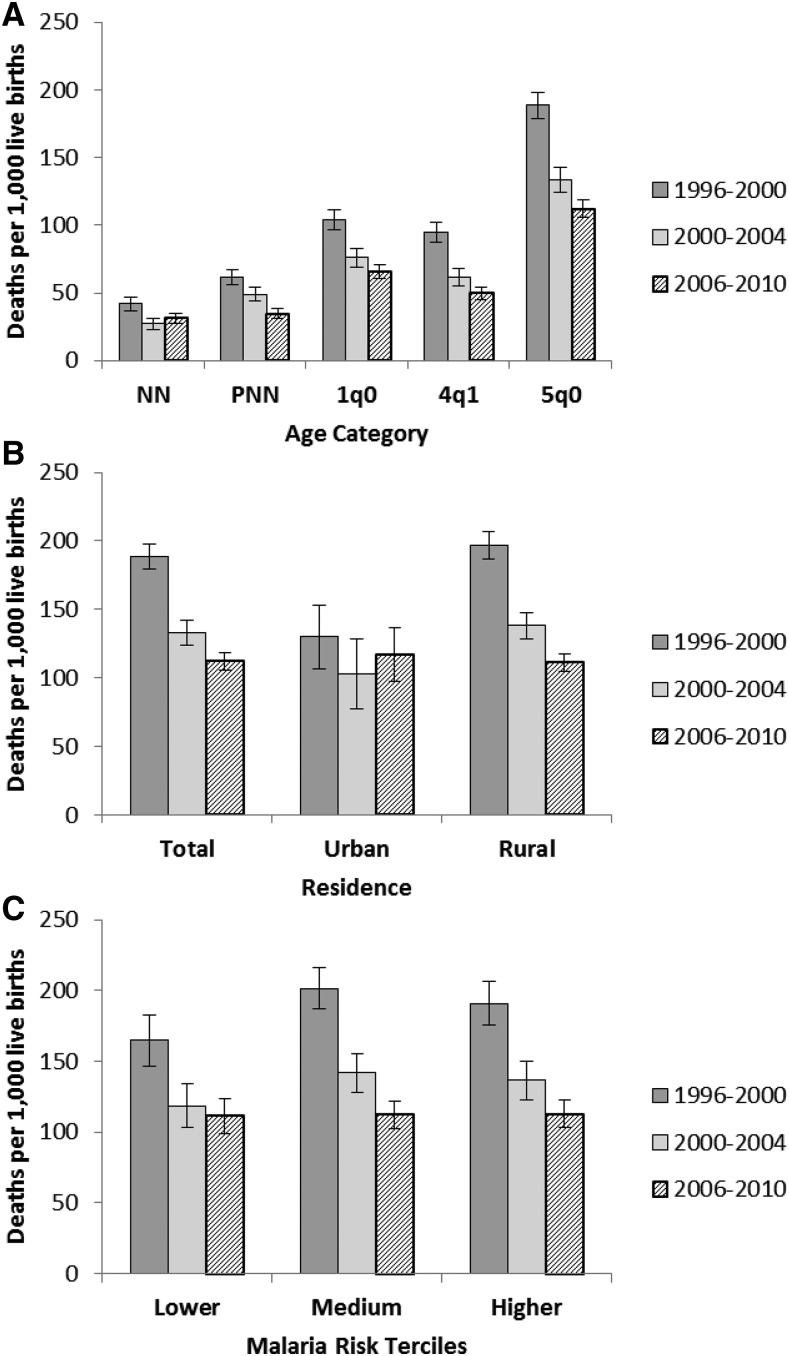
Trends in age-specific childhood mortality and all-cause under five mortality (ACCM) stratified by residence and malaria transmission risk, Malawi, 1996–2000, 2000–2004, and 2006–2010. ACCM (5q0) was estimated from the 2000, 2004, and 2010 Demographic and Health Surveys using period estimates for the 5 years before the survey. This was further disaggregated into (**A**) smaller age groups and stratified by (**B**) urban/rural residence and (**C**) malaria transmission risk areas. NN = neonatal mortality (first month) per 1,000 live births; PNN = post-neonatal mortality (age 1–11 months) per 1,000 live births; _1_q_0_ = infant mortality (first year) per 1,000 live births; _4_q_1_ = child mortality between exact age 1 and exact age 5 per 1,000 children surviving to 12 months of age; _5_q_0_ = under-five mortality per 1,000 live births.

ACCM declined substantially from 1990 to 2010 (Figure 8). Over the 20-year period, ACCM declined by over 50% and most of this decline occurred over the past 15 years (1995–2010). According to the DHS mortality estimates, significant reductions in ACCM (based on 5-year estimates) were evident during the evaluation period between 1996–2000 (189 deaths per 1,000 live births [95% CI = 179–198]) and 2000–2004 (133 [95% CI = 124–142]) (29.4% reduction) and between 2000–2004 and 2006–2010 (112 [95% CI = 106–119]) (15.8% reduction). The mortality trends by age categories demonstrated a significant decline in all age groups (Figure 9A and Supplemental Table 8), with the smallest and largest reductions in the neonatal (26% reduction) and child (47% reduction) mortality rates, respectively. Significant declines were observed in ACCM among children from rural areas, but not urban areas (Figure 9B). ACCM rates in the three malaria risk categories in the 1996–2000 period were 191, 201, and 165 deaths per 1,000 live births, in the higher, medium, and lower malaria risk terciles, respectively (Figure 9C). These had declined to 113, 112, and 111 deaths per 1,000 live births by 2006–2010, statistically significant changes from baseline in all three malaria risk terciles, although the decline was relatively larger in the medium (44.3% relative decline) and higher (40.8% relative decline) risk terciles than the lower risk tercile (32.7% relative decline).

### Contextual factors.

Contextual factors^34,35^ including socioeconomic factors, maternal and child health interventions, and meteorological factors (rainfall and temperature) were examined. The GDP per capita (PPP) remained relatively constant between 2000 and 2006 (values in the range 609–664), but then rose from 634 in 2006 to 749 in 2009, an 18.1% relative increase.^25^ Significant improvements were seen in access to an improved water source, increasing from 65.2% (95% CI = 62.1–68.2) in 2000 to 79.7% (95% CI = 77.7–81.5) in 2010; households with an improved roof, increasing from 25.8% (95% CI = 24.5–27.3) in 2006 to 35.0% (95% CI = 32.9–37.0) in 2010; electricity, increasing from 4.8% (95% CI = 3.6–6.4) in 2000 to 8.7% (95% CI = 7.6–9.9) in 2010; and telephones, increasing from 5.1% (95% CI = 3.8–6.9) in 2004 to 39.3% (95% CI = 37.6–41.1) in 2010 (Table 1). Significant improvements were seen in the percentage of women completing a primary education, increasing from 19.1% (95% CI = 17.1–21.1) in 2000 to 29.3% (95% CI = 27.8–30.8) in 2010 and the female literacy rate, increasing from 56.5% (95% CI = 54.5–58.4) in 2000 to 67.6% (95% CI = 66.3–69.0) in 2010 (Table 1). Additional indicators assessed are shown in Supplemental Table 9.

Improvements were also seen in several maternal health interventions. The proportion of women whose most recent births were protected against neonatal tetanus increased significantly from 61.0% (95% CI = 59.6–62.5) in 2000 to 68.9% (95% CI = 67.8–70.0) in 2010 and the percentage of births in a health facility also increased significantly from 55.3% (95% CI = 52.7–57.9) in 2000 to 73.2% (95% CI = 71.3–74.9) in 2010 (Table 2). However, the proportion of women who attended four or more antenatal care visits (ANC4+) as recommended by WHO decreased from 56.0% (95% CI = 54.0–57.8) to 45.5% (95% CI = 44.2–46.7) over the decade (Table 2). Additional maternal health indicators assessed are shown in Supplemental Table 9.

**Table 2:** Maternal and child health intervention coverage, Malawi, 2000 and 2010

Indicators	2000			2010			
	%	95% CI	WN	%	95% CI	WN	Relative change (%)
ANC visits 4+ (% women, most recent live birth, 0–2 years)	56.0	54.0–57.8	8,057	45.5	44.2–46.7	13,664	−18.8
Tetanus toxoid 2+ (% women, most recent live births, 0–2 years)	61.0	59.6–62.5	8,057	68.9	67.8–70.0	13,664	13.0
Delivery at a health facility (% women, live births 0–4 years)	55.3	52.7–57.9	12,201	73.2	71.3–74.9	19,697	32.4
All vaccinations (BCG, measles, DPT3, polio3) (% children 12–23 months)	70.1	67.2–72.8	2,238	80.9	78.9–82.8	3,774	15.4
Children 0–4 years had ARI symptoms in previous 2 weeks* (%)	26.7	25.3–28.1	10,559	15.4	14.5–16.3	18,013	−42.3
Children 0–4 years with ARI sought treatment (%)	26.7	24.4–29.1	2,816	65.4	62.9–67.9	2,774	144.9
Children 0–4 years with diarrhea sought treatment (%)	28.3	25.7–31.2	1,859	62.4	60.0–64.7	3,158	120.5
Children 0–4 years with diarrhea used ORS (%)	47.9	45.0–50.7	1,859	69.0	66.7–71.2	3,158	44.1
Early initiation of breastfeeding (within 1 hour of birth) (%)	72.1	70.7–73.5	11,991	67.0	66.3–67.7	19,271	−7.1
Exclusive breastfeeding in children < 6 months of age (%)	44.2	40.7–47.7	1,260	71.4	68.0–74.6	1,656	61.5
Vitamin A supplementation within past 6 months (% children 6–59 months)	70.6	69.0–72.1	9,285	85.5	84.6–86.4	16,315	21.1
Under-fives stunted (%)†	54.3	52.6–55.9	9,343	47.1	45.2–49.0	4,849	−13.3
Under-fives underweight (%)†	20.5	19.2–21.7	9,343	12.8	11.6–14.2	4,849	−37.6
Under-fives wasted (%)†	6.3	5.7–7.0	9,975	4.0	3.3–4.8	4,849	−36.5

ANC = antenatal care; ARI = acute respiratory infection; BCG = Bacillus Calmette–Guérin; CI = confidence interval; DHS = Demographic and Health Survey; DPT3 = diphtheria-tetanus-pertussis; ORS = oral rehydration solution; WHO = World Health Organization; WN = weighted number of units (denominator).

* Definition of ARI is based on data available in the 2000 survey: child had illness with cough in past 2 weeks and he/she breathed faster than usual with short, fast breaths.

† Definitions and methods per WHO reference population. Data are from the 2000 and 2010 DHS surveys. Sample sizes vary based on the population of interest corresponding to each indicator.

Coverage of several child health interventions and certain anthropomorphic measures also improved between 2000 and 2010, whereas the occurrence of some acute conditions decreased. Coverage with all vaccines (including Bacillus Calmette–Guérin [BCG], polio, measles, and diphtheria-tetanus-pertussis [DPT3]) significantly improved from 70.1% (95% CI = 67.2–72.8) to 80.9% (95% CI = 78.9–82.8) between 2000 and 2010 (Table 2 and Supplemental Table 9). Early initiation of breastfeeding worsened over the evaluation period declining from 72.1% (95% CI = 70.7–73.5) in 2000 to 67.0% (95% CI = 66.3–67.7) in 2010, whereas exclusive breastfeeding increased from 44.2% (95% CI = 40.7–47.7) in 2000 to 71.4% (95% CI = 68.0–74.6) in 2010 and Vitamin A supplementation improved from 70.6% (95% CI = 69.0–72.1) in 2000 to 85.5% (95% CI = 84.6–86.4) in 2010 (Table 2). The percentage of children less than 5 years of age with symptoms of acute respiratory infection (ARI) decreased from 26.7% (95% CI = 25.3–28.1) in 2000 to 15.4% (95% CI = 14.5–16.3) in 2010 (Table 2), whereas the percentage with diarrhea remained unchanged (Supplemental Table 9). Treatment seeking for both ARI (from 26.7% [95% CI = 24.4–29.1] to 65.4% [95% CI = 62.9–67.9]) and diarrhea (from 28.3% [95% CI = 25.7–31.2] to 62.4% [95% CI = 60.0–64.7]) improved (144.9% and 120.5% relative increases, respectively) from 2000 to 2010 (Table 2). Improvements were also seen in anthropometric measures between 2000 and 2010, as declines were noted in the proportion of children who were stunted (from 54.3% [95% CI = 52.6–55.9] to 47.1% [95% CI = 45.2–49.0]), underweight (from 20.5% [95% CI = 19.2–21.7] to 12.8% [95% CI = 11.6–14.2]), or wasted (from 6.3% [95% CI = 5.7–7.0] to 4.0% [95% CI = 3.3–4.8] (Table 2).

Among women aged 15–49 years, HIV prevalence did not change between 2004 and 2010 with an estimated 13% infected according to the DHS. Similarly, new child HIV infections remained relatively stable at 20,000 cases in 2000 and 19,000 cases in 2010.^38^ Antiretroviral (ARV) and prevention of mother-to-child transmission (PMTCT) coverage both increased during the evaluation period. HIV-positive pregnant women receiving ARVs increased from 2,179 to 33,200 between 2004 and 2009.^38^ According to UNAIDS, PMTCT coverage during pregnancy and delivery was 24% in 2009 and 53% in 2011.^39^

Meteorological data for rainfall and temperature were assessed over the evaluation period. Rainfall and temperature patterns do not suggest that any long-term climate differences existed over the period of malaria control scale-up.^4,28^ Additional meteorological analyses are presented in the accompanying article by Thomson and others.^28^

### Associations between ITN ownership and parasitemia and between ITN ownership and severe anemia.

In logistic regression models, lower household wealth status (quintile), older age (in months), not having an ITN in the home, and survey year by region (Northern, Central, and Southern) interaction were all significant predictors of malaria parasite prevalence (Table 3). Mean rainfall and mean minimum temperature anomalies did not have a significant effect on the odds of parasitemia. After adjusting for household wealth quintile, month of age, rainfall, temperature, year, and region, having at least one ITN in the household was associated with decreased odds of parasitemia in children 6–30 months of age (odds ratio [OR] = 0.81, 95% CI = 0.72–0.92).

**Table 3:** Multivariable random-effects logistic regression model of determinants of malaria parasitemia in children 6–30 months of age from sub-national Anemia and Parasitemia surveys, Malawi, 2005–2009

Parameter	Odds ratio	95% CI
Any ITNs in household	0.81*	0.72–0.92
Household wealth quintile		
Lowest (reference)	1	
Second	0.89	0.76–1.04
Middle	0.91	0.78–1.07
Fourth	0.77*	0.64–0.92
Highest	0.46*	0.37–0.58
Age (months)	1.03*	1.02–1.04
Mean rainfall anomaly, February/March	1.00	1.00–1.01
Mean minimum temperature anomaly, February/March	1.29	0.96–1.72
Year†		
2005 (reference)	1	
2007	1.95	0.97–3.90
2008	0.68	0.26–1.80
2009	0.34*	0.12–0.96
Region		
Northern region (reference)	1	
Central region	11.45*	4.10–32.03
Southern region	8.60*	2.98–24.87
Year/region interaction		
2007 × Central	0.88	0.32–2.41
2007 × Southern	0.09*	0.02–0.37
2008 × Central	0.82	0.25–2.64
2008 × Southern	0.55	0.16–1.92
2009 × Central	1.77	0.56–5.56
2009 × Southern	2.43	0.85–6.96

CI = confidence interval; ITN = insecticide-treated net.

* Significant at *P* < 0.05.

† 2006 parasitemia data were not available for this analysis.

ITN ownership was similarly protective against severe anemia in children 6–30 months of age. Lower household wealth status (quintile), younger age (in months), and not having an ITN in the home were all significant predictors of severe anemia (Table 4). Children in households in the highest wealth quintile had 45% lower odds of severe anemia than those in the lowest wealth quintile. The odds of severe anemia are highest in the youngest children, as each month of age, up to 30 months, was associated with a 3% reduction in odds of anemia. In this model, high mean minimum February/March temperature anomalies were predictive of severe anemia. Malaria infection was a strong predictor of severe anemia, with over a 4-fold increase in the odds of severe anemia if the child was parasitemic compared with if the child was not (OR = 4.25, 95% CI = 3.70–4.88). After adjusting for wealth, age, rainfall, temperature, region, year, and malaria infection, having at least one ITN in the household was associated with decreased odds of severe anemia (OR = 0.82, 95% CI = 0.72–0.94).

**Table 4:** Multivariable random-effects logistic regression model of determinants of severe anemia (hemoglobin < 8 g/dL) in children 6–30 months of age, Malawi, 2005–2009

Parameter	Odds ratio	95% CI
Any ITNs in household	0.82*	0.72–0.94
Malaria infection	4.25*	3.70–4.88
Household wealth quintile		
Lowest (reference)	1	
Second	0.85	0.72–1.01
Middle	0.76*	0.63–0.90
Fourth	0.81*	0.67–0.98
Highest	0.55*	0.44–0.70
Age (months)	0.97*	0.96–0.98
Mean rainfall anomaly, February/March	0.99*	0.99–1.00
Mean minimum temperature anomaly, February/March	1.74*	1.35–2.25
Year†		
2005 (reference)	1	
2007	1.11	0.64–1.94
2008	1.32	0.62–2.82
2009	1.09	0.45–2.66
Region		
Northern (reference)	1	
Central	0.80	0.32–2.00
Southern	0.32*	0.13–0.79
Year/region interaction		
2007 × Central	1.12	0.45–2.79
2007 × Southern	15.99*	4.73–54.10
2008 × Central	1.61	0.60–4.32
2008 × Southern	4.42*	1.60–12.23
2009 × Central	4.00*	1.41–11.37
2009 × Southern	3.88*	1.49–10.12

CI = confidence interval; ITN = insecticide-treated net.

* Significant at *P* < 0.05.

† 2006 parasitemia data were not available for this analysis.

## DISCUSSION

This evaluation found a 41% reduction in ACCM in Malawi during 2000–2010, substantial increases in household ITN ownership and use in children under five, reductions in malaria parasitemia and severe anemia, and insufficient changes in socioeconomic factors and other determinants known to influence child health to fully explain the decline in child mortality. Consequently, the evaluation indicates that malaria interventions contributed to the decline in ACCM in Malawi during 2000–2010. The association of individual ITN ownership with reduced odds of parasitemia and severe anemia further strengthens this conclusion.

The 41% decline in ACCM observed in our evaluation during 2000 and 2010 is comparable to the mortality declines over this period shown by other investigators using different mortality estimation methods (see Supplemental Figure 3 for estimates from the United Nations Interagency Group for Child Mortality Estimation^34^ and the Institute for Health Metrics and Evaluation).^35^ The magnitude of ACCM decline was greater in the medium and higher malaria risk areas compared with the lower malaria risk areas, which is consistent with what would be expected if part of the declines in ACCM were malaria-related given children in these areas had the greatest potential to benefit from increased malaria intervention coverage.^40,41^ Similarly, the relative decline in ACCM was greater in rural areas (43%) than urban areas (10%). Rural areas are where most of the malaria transmission occurs and most (84%)^42^ of the population lives. However, the largest decline (a 29% decline, representing 73% of the total decline) took place between 1996–2000 and 2000–2004, a period preceding the largest increase in malaria control intervention coverage in Malawi. However, malaria control intervention coverage was low but not insignificant between 2000 and 2004. For example, an ITN program was underway in all districts (household ownership of at least one ITN was 27% in 2004) as well as IPTp roll out (two doses of SP was 28% in 2000 and 47% in 2004). These early declines in ACCM are therefore likely due in part to the initial scaling up of malaria control interventions, along with increases in other child survival interventions and socioeconomic improvements. In the 2004–2010 period, when malaria control was intensified, mortality continued to decline.

Malaria control intervention coverage increased nationally during 2000–2010. ITNs as the primary malaria prevention tool were implemented in Malawi using a variety of delivery methods, including social marketing, commercial sector, routine distribution at ANC and EPI clinics, and mass distribution campaigns (Figure 1).^4^ A social marketing pilot in Blantyre District in 1998 was followed by subsidized nets being available in health facilities and the commercial sector in 2003. With a new long-lasting insecticidal net policy in 2008, long-lasting insecticidal nets were provided free at ANC and EPI clinics and through mass distribution campaigns.^43^ In field trials, ITNs are known to reduce ACCM by approximately 17% compared with no net use in high transmission settings.^44^ As shown in this evaluation, household ITN ownership doubled from 27% in 2004 to 57% in 2010 and ITN use among children less than 5 years of age increased from 3% to about 39% during the evaluation period. ITN use among pregnant women and the entire population reached 35% and 29%, respectively, by 2010. However, while both household ITN ownership and use remain below national and international targets, they are approaching a level where impact could be expected from both individual and community-level effects. Using malaria transmission models incorporating mosquito behavior, host availability, and survival parameters, Killeen and others estimated that even moderate (35–65%, depending on ecology) population use of ITNs can achieve community benefits (change in EIR) of ITN use beyond individual protection alone.^45^ Thus, the increase in coverage of ITNs provided individual protection to the user and likely community protection as well. Malawi used IRS as another vector control measure in addition to ITNs; however, in this national-level impact analysis, IRS was unlikely to play a significant role given only a 2% national coverage in 2010.^46^ IRS could have had important sub-national effects due to the high coverage in targeted districts.

At the time of this evaluation in 2010, Malawi had one of the highest IPTp coverage (55%) levels in sub-Saharan Africa, up from 28% in 2000.^5,47^ Thus, given the protective effect of IPTp on low birth weight,^48^ increasing IPTp coverage could have improved birth outcomes leading to reductions in neonatal mortality before the study period and continued to contribute to reductions in these outcomes over the evaluation period. However, given the minimal improvements in neonatal mortality seen here (Figure 9 and Supplemental Table 8) and the increases observed in the proportion of babies born with low birth weight (Supplemental Table 9), the effect of IPTp may have been mitigated by other contextual factors, for example a reduction in women receiving four ANC visits between 2000 and 2010 (19% relative reduction). In addition, there was an increase in the prevalence of *P. falciparum* mutations that reduce the effectiveness of SP used for IPTp in Malawi.^49^

Care seeking for fevers from formal health providers in children less than 5 years of age increased (35% in 2000 to 65% in 2010). The majority (80%) of this increase in care seeking for fever occurred in the later part of the decade between 2004 and 2010. ACTs replaced SP as the first-line antimalarial drug in 2007, midway through the evaluation period.^50,51^ Studies of ACTs have demonstrated higher rates of parasite clearance and better treatment outcomes compared with SP and cholorquine.^52,53^ Among children less than 5 years of age with fever, treatment with a first-line antimalarial increased from 23% in 2000 to 36% in 2010 and treatment with a first-line antimalarial within 24 hours increased slightly from 19% in 2000 to 24% in 2010. There are limitations to using household survey data to assess malaria case management, including problems with recall by the mothers or caretakers. In addition, the scaling up of diagnostics could have blunted the true coverage estimates of appropriate malaria case management; however, the RDT roll out occurred after the end of the evaluation period. Overall, it can be concluded that treatment of fevers increased and there was increased use of more effective antimalarial treatments over the evaluation period, which may have contributed to a decline in ACCM.

Declines were observed in malaria parasitemia and severe anemia, which lie along the causal pathway between malaria control intervention increases and reductions in malaria mortality (Figure 2). The analysis of trends in malaria parasite prevalence in children 6–35 months from the NMS revealed significant declines, as measured during the low malaria transmission season, from 61% in 2001 to 20% in 2009. In addition, the 2010 MIS showed that malaria parasitemia (43%) in children 6–59 months of age in the high malaria transmission season was also lower than the dry season estimate in 2001 (note this was in children 6–35 months of age). Two MIS conducted beyond the evaluation period in 2012 and 2014 show the malaria prevalence (by microscopy) to be 27.7% and 33.2%, respectively, in children 6–59 months of age.^54,55^ The 2012 and 2014 estimates were in the high malaria transmission season (rainy season) or just following and were lower than the 2010 MIS estimate, also conducted in the high transmission season. The prevalence in the NMS and MIS are not directly comparable due to survey design and fieldwork being conducted in different malaria transmission seasons, which poses challenges in interpreting trends in malaria parasite prevalence during and beyond the evaluation period. As a result, we included the logistic regression models to further explore the impact of malaria interventions (specifically ITNs) on malaria parasitemia and severe anemia.

In settings of high malaria endemicity, the burden of disease and the impact of malaria control are largely concentrated in infancy and early childhood.^56^ Thus, the impact of malaria control interventions on malaria-related syndromes would be expected to be larger in younger age groups (under 2 years of age) compared with those 4 or 5 years of age.^18,57^ Severe anemia in children in the subgroup 6–23 months of age decreased significantly (36% relative reduction) between 2004 and 2010 based on DHS data, whereas there was no decline in severe anemia in children 24–59 months of age. Children 6–23 months of age are at a higher risk of severe malaria-related outcomes, such as anemia and mortality, than children 24–59 months of age.^30,31^ In addition, the relative contribution of malaria to the overall anemia burden in young children would be expected to be greater in areas of medium to higher malaria transmission; thus, control of parasite transmission would be expected to reduce anemia in these populations.^19^ In line with these expectations, declines in severe anemia in the 6- to 23-month age group varied based on malaria risk zone; severe anemia declined by 46% in higher risk areas and by 44% in medium risk areas as compared with 12% in lower risk areas. Thus, the observed trends are consistent with expected changes in anemia due to malaria control interventions with respect to both age and malaria endemicity.

Appropriate considerations of contextual factors are essential for ensuring the internal and external validity of evaluations of large-scale health programs,^37^ particularly for evaluations that were conducted when rapid changes were under way in many other aspects of health services.^36^ Numerous socioeconomic factors and maternal and child health interventions were assessed for their potential impact on ACCM. Relative increases were observed in many of these factors (Tables 1 and 2 and Supplemental Table 9) including GDP (18.1% relative increase), care seeking for diarrhea (120%) and acute respiratory illness (145%), breastfeeding (61.5%), and deliveries in a health facility (32.4%), which likely contributed to reductions in ACCM. Nutritional improvements were also seen with decreases in stunting (13%), underweight (38%), and wasting (37%). Sustained high coverage of BCG, measles, DPT3, and polio3 immunizations were observed during the evaluation period with coverage at levels exceeding 80% throughout the period. Coverage with these vaccines undoubtedly saved many lives over the evaluation period, but the further increases in coverage of these vaccines are unlikely to have had a large impact on the change in mortality in children less than 5 years of age. One exception may be the increase in measles immunization coverage (83% in 2000 to 93% in 2010); measles is highly contagious, so immunization coverage levels of at least 90% need to be maintained to confer population-level protection.^58^ Also, *Haemophilus influenza (b)* and Hepatitis B immunizations were added to the EPI package, as a component of the pentavalent vaccine DPT3-HBV-Hib, during the evaluation period (2002); thus coverage of these two vaccinations rose from near-zero in 2000 to 93% in 2010, and the implementation of the Hib vaccine in particular likely contributed to the declines in ACCM. Hib is the main cause of nonepidemic bacterial meningitis in children less than 12 months of age and is also a major cause of pneumonia.^59^ It is estimated that three doses of the Hib vaccine can prevent 38–43% of the childhood meningitis mortality.^59^

Meteorological factors were considered over the evaluation period.^4,28^ Rainfall and temperature patterns do not suggest that any long-term climate differences existed over the period of malaria control scale-up that would have independently led to substantially different patterns of malaria morbidity and mortality at the end of the decade versus the start.^4^ However, analysis of meteorological data in Malawi was limited to a national level and may have missed the sub-national variation in climate factors.^28^

In addition to changes in the contextual factors highlighted here, there were improvements to the overall health system, including health financing, which could have impacted ACCM. For example, the Government of Malawi developed a Sector Wide Approach program, which included the provision of an essential healthcare package.^6^ Given that changes in fundamental determinants often act through the proximate determinants (examined here) to impact childhood morbidity and mortality, the focus here is on the proximate determinants.

To mitigate limitations inherent in plausibility based evaluation designs (in making ecologic associations between changes in malaria control intervention coverage, malaria morbidity, and ACCM at a national level), multivariable models were used to assess the association of one malaria intervention (ITN ownership) with malaria parasitemia, severe anemia, and child survival. In sub-national district analysis between 2005 and 2009, household ownership of ITNs was protective against malaria parasitemia and severe anemia in children 6–30 months of age, controlling for meteorological factors, geographic location, household wealth, and child’s age; odds of parasitemia were 19% lower and odds of severe anemia were 18% lower in children living in households with at least one ITN compared with those without ITNs.

We also found that household ITN ownership was associated with increased child survival using a Cox proportional hazards model and a district-level platform model presented in the accompanying paper by Florey and others.^16^ It should be noted that the Cox proportional hazards model assesses individual level associations, which could be influenced by residual confounding and may not translate directly to population level impact. However, given there are numerous controlled studies that have assessed the association between ITNs and malaria morbidity and mortality,^8,44^ it was important to assess the association in Malawi under programmatic conditions where distribution of ITNs was not under the control of a study, but relied on the routine distribution systems operating in country (retail, social marketing, ANC/EPI routine distribution, and mass campaigns).

Malawi has made progress in improving coverage of malaria prevention and treatment interventions during the evaluation period. Part of the decline in all-cause mortality, particularly the declines earlier in the evaluation period could be due to the improvements in the coverage of socioeconomic factors and other child health interventions as well as the initial expansion of malaria control interventions. In addition, it is likely that the decline in all-cause mortality among children less than 5 years of age was in part due to a reduction in malaria-specific mortality, particularly later in the evaluation period. Multivariable models support this claim; ITN ownership is associated with improved child survival in Malawi.^16^ In addition, ITN ownership was found to be protective against malaria morbidity measures (severe anemia and parasitemia in children 6–30 months of age). Taken together this evidence suggests that malaria control interventions in Malawi likely contributed to reductions in ACCM during 2000–2010. Reductions in ACCM will likely continue as intervention coverage intensifies and the overall malaria control program increases efficiencies in service delivery.

## Supplementary Material

Supplemental Tables.
