# Supplementary material for: Malaria Control Interventions Contributed to Declines in Malaria Parasitemia, Severe Anemia, and All-Cause Mortality in Children Less Than 5 Years of Age in Malawi, 2000–2010
Source: Am J Trop Med Hyg. 2017 Sep 27;97(3 Suppl):76–88. doi: 10.4269/ajtmh.17-0203 (PMC5619935; doi:10.4269/ajtmh.17-0203)
Supplement: Supplementary file 1 [file tpmd170203.SD1.pdf]

The following are supplemental materials and will be published online only

doi:10.4269/ajtmh.17-0203.s001

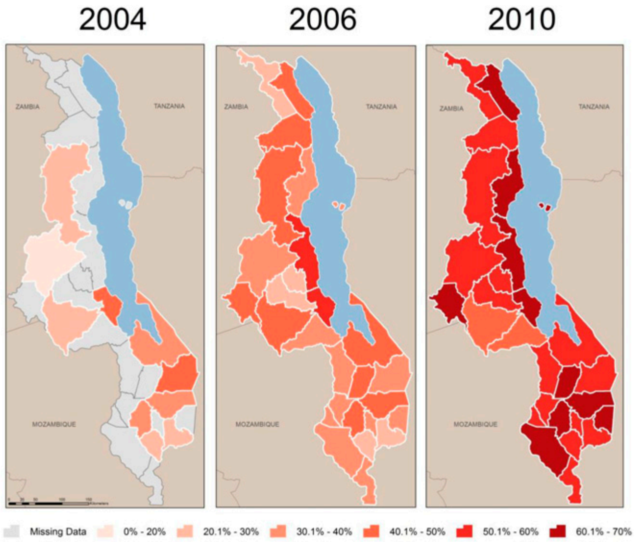

Supplemental Figure 1. Household ownership of insecticide-treated nets (ITNs) by district, Malawi, 2004–2010. Household ITN ownership was mapped by district from the 2004 and 2010 Demographic and Health Survey and the 2006 Multiple Indicator Cluster Survey.

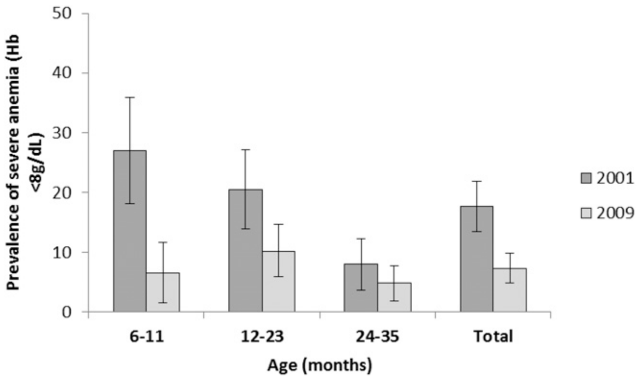

Supplemental Figure 2. Severe anemia (hemoglobin < 8 g/dL) prevalence in children 6–35 months by age, Malawi, 2001 and 2009. Anemia prevalence data are from the 2001 and 2009 National Micronutrient Surveys conducted in the low malaria transmission season for children 6–35 months of age (total) and further disaggregated by age.

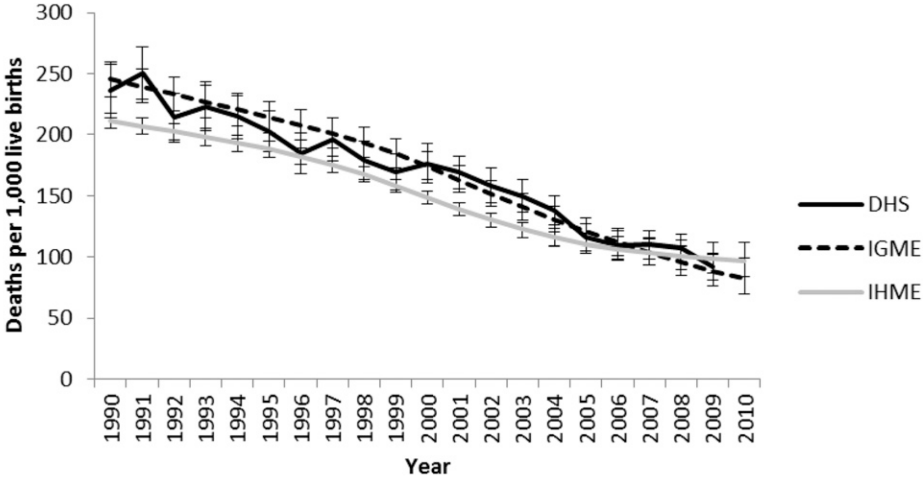

Supplemental Figure 3. Annual estimates were calculated for all-cause under-five mortality from 1990 to 2010 using the 2000 and 2010 Demographic and Health Survey (DHS). United Nations Interagency Group for Child Mortality Estimation (IGME) (accessed April 23, 2015)[34] and Institute for Health Metrics and Evaluation (IHME)[35] annual estimates of under-five mortality are also shown for comparison. Methods for calculating the IGME and IHME mortality estimates are described elsewhere.[34,35]

The following are supplemental materials and will be published online only

Supplemental Table 1

Malaria indicators and data sources used in this evaluation

Indicator	Data source
Proportion of households with at least one ITN	2004, 2010 DHS; 2006 MICS
Proportion of children under 5 years old who slept under an ITN the previous night	2000, 2004, 2010 DHS; 2006 MICS
Proportion of pregnant women who slept under an ITN the previous night	2000, 2004, 2010 DHS; 2006 MICS
Proportion of population who slept under an ITN the previous night	2004, 2010 DHS
Proportion of households sprayed by IRS in the last 12 months	2010 DHS
Proportion of women who received two doses of SP for malaria during their last pregnancy	2000, 2004, 2010 DHS; 2006 MICS
Proportion of children under 5 years old with fever in the last 2 weeks for whom advice or treatment was sought	2000, 2004, 2010 DHS
Proportion of children under 5 years old with fever in the last 2 weeks for whom advice or treatment was sought within 24 hours	2000, 2004, 2010 DHS
Proportion of children under 5 years old with fever in last 2 weeks who received any antimalarial treatment	2000, 2004, 2010 DHS; 2006 MICS
Proportion of children under 5 years old with fever in last 2 weeks who received first-line treatment according to national policy	2000, 2004, 2010 DHS; 2006 MICS
Proportion of children under 5 years old with fever in last 2 weeks who received first-line treatment according to national policy within 24 hours from onset of fever	2000, 2004, 2010 DHS; 2006 MICS
Proportion receiving first-line treatment, among children under 5 years old with fever in the last 2 weeks who received any antimalarial drugs	2000, 2004, 2010 DHS; 2006 MICS
Severe anemia prevalence: proportion of children aged 6–59 months with a hemoglobin measurement of < 8 g/dL	2004 and 2010 DHS; 2001 and 2009 National Micronutrient Surveys (6–35 months); 2005–2009 sub-national Anemia and Parasitemia surveys (6–30 months)
Parasite Prevalence: proportion of children aged 6–59 months with malaria infection [all surveys used microscopy]	2010 MIS; 2001 and 2009 National Micronutrient Surveys (6–35 months); 2005–2009 sub-national Anemia and Parasitemia surveys (6–30 months)
All-cause under 5 mortality rate	2000, 2004, 2010 DHS

DHS = Demographic and Health Survey; ITN = insecticide-treated net; IRS = indoor residual spraying; MICS = Multiple Indicator Cluster Survey; MIS = Malaria Indicator Survey; SP = sulfadoxine-pyrimethamine.

Supplemental Table 2

ITN use among children less than 5 years of age by background characteristics, Malawi, 2000–2010

	2000			2004			2006			2010			Relative change (%)*
	%	95% CI	WN	%	95% CI	WN	%	95% CI	WN	%	95% CI	WN	
Sex													
Male	2.8	2.2–3.5	5,225	14.3	12.8–16.0	5,195	25.1	23.4–26.9	11,368	38.6	37.0–40.3	9,514	1,279
Female	2.7	2.1–3.6	5,334	15.2	13.6–17.0	5,344	24.2	22.6–26.0	11,626	40.2	38.5–41.9	9,905	1,389
Residence													
Urban	11.5	9.2–14.2	1,358	30.2	25.5–35.4	1,420	42.3	38.4–46.3	3,366	48.4	43.6–53.2	2,634	321
Rural	1.5	1.1–1.9	9,201	12.4	11.1–13.8	9,119	21.6	20.5–22.8	19,628	38.0	36.5–39.5	16,785	2,433
Wealth quintile													
Lowest	0.6	0.3–1.1	2,311	6.4	4.8–8.3	2,090	14.6	13.0–16.4	5,112	28.8	26.6–31.2	4,344	4,700
Second	0.9	0.5–1.5	2,144	9.2	7.7–11.0	2,294	19.9	17.7–22.3	4,686	35.1	32.9–37.4	4,200	3,800
Middle	2.2	1.2–4.1	1,965	12.1	10.3–14.2	2,358	24.1	21.9–26.5	4,736	41.5	39.0–44.0	4,158	1,786
Fourth	2.4	1.6–3.5	2,120	16.7	14.4–19.2	2,071	25.5	23.2–28.1	4,243	42.1	39.5–44.8	3,587	1,654
Highest	8.2	6.6–10.1	2,007	33.8	29.4–38.5	1,726	41.8	38.7–45.1	4,217	54.0	50.8–57.2	3,130	559
Mother's education													
None	0.8	0.5–1.3	3,388	7.3	6.1–8.7	2,557	18.1	15.4–21.3	5,614	32.4	29.8–35.0	3,231	3,950
Primary	2.0	1.5–2.7	6,453	15.3	13.7–17.0	6,219	23.6	22.3–25.0	14,875	39.7	38.2–41.3	12,250	1,885
Secondary+	18.4	14.9–22.4	718	33.8	29.3–38.5	1,025	46.2	42.9–49.5	2,442	56.6	53.2–60.0	2,563	208
Missing	–			10.1	7.3–13.8	737	23.6	12.9–39.2	53	21.1	18.1–24.6	1,376	–
Total	2.8	2.2–3.4	10,599	14.8	13.4–16.3	10,539	24.7	23.1–26.3	22,994	39.4	38.0–40.8	19,420	1,307

CI = confidence interval; DHS = Demographic and Health Survey; ITN = insecticide-treated net; MICS = Multiple Indicator Cluster Survey; WN = weighted number of units (denominator). Data are from the 2000, 2004, and 2010 DHS and 2006 MICS surveys.

* Relative change between the 2000 and 2010 estimates. Sample sizes vary based on the population of interest corresponding to each indicator.

The following are supplemental materials and will be published online only

Supplemental Table 3

ITN use among pregnant women by background characteristics, Malawi, 2000–2010

	2000			2004			2006*			2010			Relative change (%)†
	%	95% CI	WN	%	95% CI	WN	%	95% CI	WN	%	95% CI	WN	
Residence													
Urban	9.6	5.6–16.0	206	29.8	21.9–39.2	183	40.3	24.4–58.6	85	43.6	32.2–55.7	248	354
Rural	1.6	1.0–2.5	1,362	12.4	10.4–14.8	1,222	22.2	17.7–27.5	453	34.1	31.5–36.8	1,838	2,031
Wealth quintile													
Lowest	0.7	0.2–2.4	335	6.0	3.7–9.6	247	14.3	7.2–26.3	87	22.5	18.4–27.2	416	3,114
Second	0.3	0.0–1.8	288	9.6	6.8–13.5	319	16.7	9.7–27.4	141	28.6	24.2–33.6	478	9,433
Middle	2.7	1.4–5.1	309	12.6	9.3–16.8	342	16.8	9.4–28.2	120	38.1	33.2–43.3	481	1,311
Fourth	2.5	1.2–5.0	344	17.0	12.2–23.1	284	47.9	30.3–66.0	110	42.2	36.4–48.2	370	1,588
Highest	7.4	4.3–12.5	289	32.6	25.1–41.1	213	32.7	18.4–51.2	80	48.5	39.2–58.0	341	555
Woman's education													
None	0.7	0.2–2.1	426	8.6	5.8–12.4	309	19.4	12.1–29.8	111	31.7	25.5–38.6	294	4,429
Primary	2.0	1.3–3.2	1,032	14.5	12.0–17.4	927	26.3	20.3–33.4	379	33.0	30.1–36.0	1,479	1,550
Secondary +	15.9	9.2–26.0	110	26.7	19.0–36.1	169	28.9	14.9–48.6	47	49.3	42.4–56.1	312	210
Missing	–			–			–			44.2	8.8–86.6	2	–
Total	2.6	1.8–3.8	1,568	14.7	12.5–17.1	1,405	25.1	19.9–31.1	538	35.2	32.6–38.0	2,086	1,254

CI = confidence interval; DHS = Demographic and Health Survey; MICS = Multiple Indicator Cluster Survey; WN = weighted number of units (denominator).

* Based on pregnant women who had a birth in the past 2 years (2006). The other surveys are based on pregnant women who had a birth in the past 5 years. Data is from the 2000, 2004, and 2010 DHS and 2006 MICS surveys. Sample sizes vary based on the population of interest corresponding to each indicator.

† Relative change is from 2000 to 2010.

Supplemental Table 4

Use of intermittent preventive treatment during pregnancy* by background characteristics, Malawi, 2000–2010

	2000			2004			2006			2010			Relative change (%)†
	%	95% CI	WN	%	95% CI	WN	%	95% CI	WN	%	95% CI	WN	
Residence													
Urban	31.5	27.4–35.9	644	53.8	48.8–58.7	583	55.1	50.2–59.9	1,507	55.9	51.0–60.7	1,138	77.5
Rural	27.8	26.1–29.4	4,419	45.4	43.4–47.4	4,021	45.3	43.7–47.0	9,045	54.8	53.2–56.5	6,586	97.1
Wealth quintile													
Lowest	25.7	22.7–29.0	1,118	42.1	38.2–46.0	919	41.8	38.4–45.2	2,442	52.7	49.5–55.8	1,669	105.1
Second	27.2	24.1–30.4	1,023	42.6	39.1–46.3	1,111	44.2	41.1–47.4	2,225	54.3	51.5–57.2	1,669	99.6
Middle	28.9	25.6–32.5	915	46.2	42.7–49.7	1,001	47.0	44.0–50.0	2,164	56.9	53.8–59.9	1,689	96.9
Fourth	29.8	26.9–32.8	1,060	47.8	44.1–51.5	871	48.1	44.2–52.0	1,899	56.2	52.8–59.6	1,409	88.6
Highest	29.9	26.5–33.4	943	57.0	52.9–61.0	701	54.6	51.0–58.1	1,822	55.1	51.0–59.1	1,288	84.3
Woman's education													
None	25.9	23.3–28.6	1,507	39.7	36.1–43.4	1,110	39.4	36.7–42.2	2,407	52.5	48.6–56.4	1,249	102.7
Primary	28.3	26.4–30.4	3,172	47.1	44.9–49.4	2,960	46.7	44.9–48.5	6,912	54.7	52.8–56.6	5,236	93.3
Secondary +	36.7	31.0–42.8	383	57.0	51.9–61.9	534	60.8	55.2–66.2	1,232	58.9	55.2–62.5	1,239	60.5
Total	28.2	26.7–29.8	5,063	46.5	44.6–48.3	4,604	46.7	45.0–48.4	10,552	55.0	53.4–56.6	7,724	95.0

CI = confidence interval; DHS = Demographic and Health Survey; MICS = Multiple Indicator Cluster Survey; SP = sulfadoxine-pyrimethamine; WN = weighted number of units (denominator).

* Percentage of women age 15–49 with a live birth in the 2 years preceding the survey who received at least two doses of SP/Fansidar without regard to the source of SP for prevention of malaria during last pregnancy. Data are from the 2000, 2004, and 2010 DHS and 2006 MICS surveys. Sample sizes vary based on the population of interest corresponding to each indicator.

† Relative change is from 2000 to 2010.

The following are supplemental materials and will be published online only

SUPPLEMENTAL TABLE 5

Care seeking* for fever in children less than 5 years of age, by background characteristics, Malawi, 2000–2010

	2000			2004			2006			2010			
	%	95% CI	WN	%	95% CI	WN	%	95% CI	WN	%	95% CI	WN	Relative change (%)†
Sex													
Male	34.8	32.2–37.6	2,179	42.6	39.7–45.5	1,793	43.4	41.1–45.8	3,887	65.4	63.0–67.7	3,161	87.9
Female	35.7	33.1–38.4	2,215	40.0	37.0–43.2	1,837	42.5	40.0–45.0	4,102	65.3	63.2–67.4	3,053	82.9
Residence													
Urban	45.8	38.1–53.6	434	56.8	50.4–63.1	401	54.6	48.0–61.1	993	69.2	64.3–73.7	786	51.1
Rural	34.1	31.9–36.4	3,961	39.4	36.9–41.8	3,230	41.3	39.6–43.0	6,997	64.8	63.0–66.6	5,428	90.0
Wealth quintile													
Lowest	33.9	30.1–37.9	1,032	35.7	31.5–40.1	757	40.3	36.2–44.4	1,899	61.1	57.6–64.5	1,397	80.2
Second	34.3	30.6–38.2	884	41.0	36.6–45.5	894	42.8	39.3–46.3	1,685	61.7	58.2–65.1	1,357	79.9
Middle	34.4	30.4–38.6	783	38.7	34.7–43.0	830	40.0	37.0–43.1	1,735	69.0	65.9–71.9	1,469	100.6
Fourth	31.7	27.8–35.9	944	44.7	40.2–49.3	676	45.2	41.9–48.4	1,530	64.8	60.9–68.4	1,127	104.4
Highest	44.2	38.6–49.8	745	50.4	44.5–56.3	474	49.2	44.8–53.7	1,141	72.7	68.0–77.0	865	64.5
Mother's education													
None	30.5	27.3–33.9	1,404	34.5	31.0–38.2	911	40.8	36.4–45.3	1,985	59.5	55.3–63.6	952	95.1
Primary	36.0	33.5–38.7	2,768	42.0	39.3–44.9	2,379	41.7	39.9–43.4	5,206	65.2	63.2–67.2	4,429	81.1
Secondary +	56.0	44.0–67.3	223	54.1	46.8–61.2	340	57.1	50.5–63.4	784	73.0	68.5–77.1	833	30.4
Nonstandard curriculum	–			–			42.5	21.5–66.6	16	–			-
Total	35.3	33.1–37.5	4,394	41.3	38.9–43.7	3,630	43.0	41.0–44.9	7,990	65.4	63.6–67.1	6,214	85.3

CI = confidence interval; DHS = Demographic and Health Survey; MICS = Multiple Indicator Cluster Survey; WN = weighted number of units (denominator).

* Care seeking refers to seeking advice or treatment of fever from a public or private health professional or from a pharmacy, including health surveillance assistants (HSAs)/community health workers, but excluding shops and traditional healers. Data from 2000, 2004, 2010 DHS and 2006 MICS. Sample sizes vary based on the population of interest corresponding to each indicator.

† Relative change from 2000 to 2010.

SUPPLEMENTAL TABLE 6

Among children less than 5 years of age with recent fever who received antimalarial treatment proportion who received recommended treatment*, by background characteristics, Malawi, 2000–2010

	2000			2004			2006			2010			
	%	95% CI	WN	%	95% CI	WN	%	95% CI	WN	%	95% CI	WN	Relative change (%)†
Sex													
Male	84.6	80.5–87.9	586	83.7	79.8–87.0	515	79.0	75.2–82.3	1,003	81.8	78.8–84.4	1,369	–3.3
Female	87.4	83.9–90.2	603	80.7	76.1–84.5	517	83.3	80.0–86.2	987	86.0	83.1–88.5	1,328	–1.6
Residence													
Urban	81.8	71.7–88.9	146	81.7	69.2–89.8	168	78.9	71.1–85.1	319	80.8	71.3–87.8	335	–1.2
Rural	86.6	83.6–89.1	1,043	82.3	79.3–84.9	864	81.5	78.8–84.0	1,671	84.3	82.0–86.3	2,362	–2.7
Wealth quintile													
Lowest	88.8	81.2–93.6	255	83.6	75.6–89.4	176	83.2	75.6–88.8	374	86.0	81.8–89.4	571	–3.2
Second	90.4	85.5–93.8	226	85.0	78.9–89.5	235	83.1	78.5–86.9	417	87.7	84.0–90.7	577	–3.0
Middle	83.0	76.5–88.0	206	79.6	73.4–84.6	211	81.0	74.5–86.1	434	86.3	82.6–89.3	659	4.0
Fourth	91.5	86.6–94.7	243	83.9	77.3–88.8	223	79.8	72.5–85.6	406	80.7	75.5–84.9	492	–11.8
Highest	76.5	69.3–82.4	258	78.3	69.6–85.1	187	78.3	72.3–83.3	359	75.1	69.2–80.3	399	–1.8
Mother's education													
None	88.1	82.1–92.3	335	82.6	76.1–87.7	196	82.4	74.4–88.3	427	84.6	78.8–89.0	373	–4.0
Primary	85.9	82.4–88.8	758	83.2	79.7–86.2	695	81.4	78.1–84.2	1,279	85.1	82.7–87.1	1,935	–0.9
Secondary +	79.1	62.8–89.4	96	76.5	67.4–83.7	141	78.2	69.5–84.9	279	77.2	70.2–83.0	388	–2.4
Nonstandard curriculum	–			–			71.7	29.5–93.9	5	–			–
Total	86.0	83.2–88.4	1,189	82.2	79.1–84.9	1,032	81.1	78.6–83.4	1990	83.9	81.7–85.9	2,696	–2.4

ACT = artemisinin-based combination therapy; CI = confidence interval; DHS = Demographic and Health Survey; MICS = Multiple Indicator Cluster Survey; SP = sulfadoxine-pyrimethamine; WN = weighted number of units (denominator).

* SP was the recommended antimalarial in 2000, 2004, and 2006; ACT (AL) in 2010. Data from 2000, 2004, 2010 DHS and 2006 MICS. Sample sizes vary based on the population of interest corresponding to each indicator.

† Relative change from 2000 to 2010.

The following are supplemental materials and will be published online only

Supplemental Table 7

Severe anemia (hemoglobin < 8 g/dL) prevalence in children 6–59 months of age, by background characteristics, Malawi, 2004 and 2010

	2004			2010			
	%	95% CI	WN	%	95% CI	WN	Relative change (%)*
Sex							
Male	11.6	9.6–14.0	1,060	9.1	7.7–10.8	2,224	–21.6
Female	9.7	7.8–12.0	1,113	8.2	6.7–10.0	2,291	–15.5
Residence							
Urban	4.9	2.4–9.8	231	6.8	4.2–10.9	636	38.8
Rural	11.3	9.7–13.2	1,942	9.0	7.8–10.4	3,879	–20.4
Wealth quintile							
Lowest	13.4	10.2–17.5	414	11.4	8.9–14.5	819	–14.9
Second	14.2	10.6–18.8	490	9.3	7.2–12.0	1,038	–34.5
Middle	9.4	6.8–12.7	537	9.3	7.0–12.2	997	–1.1
Fourth	10.0	7.1–13.8	454	7.9	5.8–10.6	833	–21.0
Highest	3.8	2.0–7.1	280	5.3	3.6–7.8	828	39.5
Mother's education							
None	14.3	11.0–18.4	533	10.7	8.0–14.2	767	–25.2
Primary	10.2	8.5–12.1	1,283	8.9	7.5–10.4	2,900	–12.7
Secondary +	10.6	6.6–16.6	200	6.4	4.2–9.7	619	–39.6
Missing	2.1	0.8–5.0	158	5.8	3.2–10.0	229	–
Total	10.6	9.1–12.4	2,173	8.7	7.6–10.0	4,515	–17.9

CI = confidence interval; DHS = Demographic and Health Survey; MICS = Multiple Indicator Cluster Survey; WN = weighted number of units (denominator). Data are from the 2004 and 2010 DHS surveys. Sample sizes vary based on the population of interest corresponding to each indicator. The 2000 DHS and 2006 MICS did not collect information on anemia.

* Relative change from 2004 to 2010.

Supplemental Table 8

Trends in age-specific childhood mortality, Malawi, 1996–2000, 2000–2004, and 2006–2010

	1996–2000		2000–2004		2006–2010		
	Estimate	95% CI	Estimate	95% CI	Estimate	95% CI	Relative change (%)*
Neonatal mortality (NN)	42	37–47	27	23–31	31	28–35	–26.2
Postneonatal mortality (PNN)	62	56–67	49	44–54	35	31–39	–43.5
Infant mortality (1q0)	104	96–111	76	69–83	66	61–71	–36.5
Child mortality (4q1)	95	87–102	62	55–68	50	45–54	–47.4
Under-five mortality (5q0)	189	179–198	133	124–142	112	106–119	–40.7

CI = confidence interval; DHS = Demographic and Health Survey; Age-specific all-cause mortality (per 1,000 live births) for 5-year periods preceding the survey is shown. Data are from the 2000, 2004, and 2010 DHS surveys.

* Relative change from 1996–2000 to 2006–2010 periods. NN = neonatal mortality (first month), per 1,000 live births; PNN = post-neonatal mortality (age 1–11 months), per 1,000 live births; ${}_1q_0$ = infant mortality (first year), per 1,000 live births; ${}_4q_1$ = child mortality between exact age 1 and exact age 5, per 1,000 children surviving to 12 months of age; ${}_5q_0$ = under-five mortality, per 1,000 live births.

Supplemental Table 9

Additional household attributes and maternal and child health intervention coverage, Malawi, 2000 and 2010

Indicators	2000			2010				
	%	95% CI	WN	%	95% CI	WN	Relative change (%)**	Sig
Time to water source < 15 minutes (% households)	33.4	31.2–35.7	14,213	34.7	32.9–36.6	24,825	3.9	NS
Modern floor material (not earth/sand/dung), (% households)	18.8	16.5–21.4	14,213	23.3	21.3–25.3	24,825	23.9	NS
Births in any high-risk fertility category (%)*	57.3	56.2–58.5	12,201	56.6	55.4–57.7	19,697	–1.2	NS
Births with unavoidable fertility risk (%)†	16.5	15.7–17.3	12,201	14.4	13.7–15.1	19,697	–12.7	S
BCG (% children 12–23 months)	92.4	90.7–93.8	2,238	97.2	96.4–97.8	3,774	5.2	S
DPT3/DPT3-HBV-Hib (% children 12–23 months)	84.2	81.8–86.4	2,238	93.0	91.7–94.2	3,774	10.5	S
Polio3 (% children 12–23 months)	79.8	77.2–82.2	2,238	85.6	83.9–87.2	3,774	7.3	S
Measles (% children 12–23 months)	83.2	80.9–85.3	2,238	93.0	91.8–94.0	3,774	11.8	S
Children 0–4 years with diarrhea in previous 2 weeks (%)	17.6	16.7–18.6	10,559	17.5	16.8–18.3	18,013	–0.6	NS
Small/very small size at birth (mother's estimate) (%)	16.6	15.7–17.6	12,201	15.5	14.8–16.3	19,697	–6.6	NS
Low birth weight < 2,500 g (%)	4.9	4.4–5.5	12,201	8.2	7.6–8.8	19,697	67.3	S

BCG = Bacillus Calmette–Guérin; DPT3 = diphtheria-tetanus-pertussis; HBV-Hib = *Haemophilus influenza (b)* and Hepatitis B, CI = confidence interval; DHS = Demographic and Health Survey; Sig. = Statistical significance; WN = weighted number of units (denominator). Statistics with nonoverlapping 95% CIs are considered significantly different changes. NS denotes no statistically significant change and S denotes statistically significant change. Data are from the 2000 and 2010 DHS surveys. Sample sizes vary based on the population of interest corresponding to each indicator.

* Births to women < 18 and > 34 and births < 2 years apart.

† First order births to women between the ages of 18 and 34.

** Relative change from 2000 to 2010.
